# Parkinsonism in Spinocerebellar Ataxia

**DOI:** 10.1155/2015/125273

**Published:** 2015-03-19

**Authors:** Hyeyoung Park, Han-Joon Kim, Beom S. Jeon

**Affiliations:** Departments of Neurology and Movement Disorder Center, College of Medicine, Seoul National University Hospital, Seoul National University, Seoul 110-744, Republic of Korea

## Abstract

Spinocerebellar ataxia (SCA) presents heterogeneous clinical phenotypes, and parkinsonism is reported in diverse SCA subtypes. Both levodopa responsive Parkinson disease (PD) like phenotype and atypical parkinsonism have been described especially in SCA2, SCA3, and SCA17 with geographic differences in prevalence. SCA2 is the most frequently reported subtype of SCA related to parkinsonism worldwide. Parkinsonism in SCA2 has unique genetic characteristics, such as low number of expansions and interrupted structures, which may explain the sporadic cases with low penetrance. Parkinsonism in SCA17 is more remarkable in Asian populations especially in Korea. In addition, an unclear cutoff of the pathologic range is the key issue in SCA17 related parkinsonism. SCA3 is more common in western cohorts. SCA6 and SCA8 have also been reported with a PD-like phenotype. Herein, we reviewed the epidemiologic, clinical, genetic, and pathologic features of parkinsonism in SCAs.

## 1. Introduction

Spinocerebellar ataxia (SCA) is a progressive, autosomal dominant neurodegenerative disorder which affects the cerebellum and its connected structures. Even though ataxia is a main feature in most cases, clinically there are various phenotypes even in the same SCA subtype which shows numerous clinical features related to the brainstem and spinal cord with or without ataxia [1]. Many extrapyramidal symptoms including parkinsonism are also seen in diverse SCA subtypes.

In the literature, SCA3 or Machado-Joseph disease (MJD) was the first genetically confirmed SCA subtype in a patient with the levodopa-responsive Parkinson disease (PD) like phenotype, although the symptoms of this patient did not exactly resemble idiopathic PD [2]. Since then, many SCA subtypes, such as SCA2 [3–15], SCA6 [16–18], SCA8 [19], and SCA17 [20–22], have been described as both levodopa-responsive PD and atypical parkinsonism.

We reviewed the clinical features of parkinsonism in SCAs and discuss the various characteristics from genetic background to pathology. Herein, we focused especially on SCA2 and SCA17 which have been frequently described (Table 1).

## 2. SCA2

### 2.1. Epidemiology

SCA2 is the most frequently reported subtype of SCA related to parkinsonism worldwide. The first report of a SCA2 gene mutation with parkinsonism was in a large Chinese family, presenting as familial progressive supranuclear palsy (PSP) and PD [3]. The authors evaluated 58 family members in four linear generations. There were a total of 11 affected members and 6 of them were alive. Three of the four family members with a clinical PD phenotype showed levodopa responsiveness and one of them had levodopa induced dyskinesia. The fourth member developed mild ataxia later in the course of the disease. Their trinucleotide repeats (TNR) expansion numbers were 35 and 36. One patient with a PSP phenotype had a repeat number of 33. Three patients with ataxia had a younger age at onset with a longer repeat number (*N* = 43).

The prevalence varies depending on ethnicity and family history. In the European population, SCA2 is not a rare cause of familial parkinsonism. Among 164 French families with autosomal dominant parkinsonism (ADP), three families with nine patients had SCA2 mutations (2%) [23]. All of them had levodopa responsiveness without cerebellar signs. The SCA2 patients seemed to be significantly less asymmetrical and less rigid than patients with mutations in other genes. They also required less levodopa and had fewer fluctuations than other genetic causes [23]. In a Brazilian study, the prevalence was 3.4% in familial parkinsonism patients. Intrafamilial, phenotypic homogeneity was a characteristic feature in these Brazilian SCA2 kindred [14]. Modoni et al. [24] detected SCA2 mutations in approximately 1% of Italian familial parkinsonism patients. The patients were tremor-dominant and levodopa-responsive with an abnormal FP-CIT positron emission tomography (PET) scan. In the USA, there were two studies on familial parkinsonism in a mixed population; and SCA2 mutations at a rate of 1.5% [6] and 0.88% [13] were found. In sporadic PD patients, studies failed to find SCA2 mutations in Italy [25] and Serbia [7]. In Asians, the frequencies differ even within the same ethnic group, and the frequency of SCA2 mutations varied from less than 2% [13] to up to 8.7% [4] in familial parkinsonism. The marked difference in prevalence could possibly be explained by the difference in selected cohorts [26]. In contrast to the western studies, sporadic PD patients also showed SCA2 mutations in Asian studies although the prevalence of SCA2 mutations in sporadic form (0.4–2.2%) was lower than that of familial cases [26–29]. In Singapore, the SCA2 mutation frequency in a Chinese population was 2.2% in early onset sporadic PD patients, and those cases had an expanded allele of 36 CAG repeats [26]. In Taiwan [11] and in mainland China [27], expanded CAG repeats in the SCA2 locus were found in 0.4% and 0.5% of sporadic PD patients, respectively. In Korea, among a total of 603 parkinsonian patients (468 with PD and 135 with multiple system atrophy-parkinsonian phenotype (MSA-P)), two patients with a PD phenotype and one patient with a MSA-P phenotype were identified to have an expanded SCA2 allele (0.5% with PD phenotype and 0.7% with MSA-P phenotype) [28].

### 2.2. Clinical Features

Usual manifestations of SCA2 mutations are cerebellar ataxia, dysarthria, tremor, hypoactive deep tendon reflexes, peripheral neuropathy, and slow saccadic eye movements [30]. Clinical features of parkinsonism in SCA2 varied from sporadic PD mimicking [5, 6, 9, 10] to a MSA phenotype [28]. Most parkinsonian cases with SCA2 had normal saccadic movement which was a distinctive feature of SCA2. The onset age of parkinsonism was not different between familial and sporadic cases (29 to 70 years old) in many cohort studies [6, 10, 13, 23, 26–29]. Patients with a PD phenotype have shown a good levodopa response; and some of them reported typical dyskinesia and motor fluctuation [6, 11, 12, 14, 23]. Two patients with a MSA phenotype were diagnosed with MSA-P; one of them had parkinsonism and autonomic failure but no cerebellar symptoms including ataxia. The other presented with parkinsonism and autonomic symptoms initially, but ataxia developed after 2 years of follow-up. Both of them showed minimal or no improvement in parkinsonian symptoms from the levodopa treatment [28, 29]. These MSA phenotype patients showed mild cerebellar atrophy on the brain MRI and decreased striatal uptake on dopamine transporter imaging. Other previous DAT-imaging data for SCA2 related parkinsonian patients have shown nigrostriatal dopaminergic damage similar to that of PD with a rostrocaudal gradient [4, 5, 24, 31]. Asymptomatic carriers also have shown a reduction of CIT binding in the putamen [28]. Hence, dopamine transporter imaging may be a useful method to evaluate nigrostriatal dopaminergic damage in the presymptomatic stage in mutation carriers of SCA2.

### 2.3. Genetic Characterization

In SCA2, 31 or fewer CAG repeats are regarded as normal alleles [32, 33]. In a Korean study [28], 30 patients with ataxia had a CAG expansion of 38 to 51, whereas three patients with parkinsonism were found with 32, 34, and 35 repeats. Of great interest is that all SCA2 parkinsonian patients were sporadic cases, emphasizing the need to screen for SCA2 mutations even in patients with nonfamilial parkinsonism [28]. Previous reports have also shown that SCA2-related parkinsonism carries low to intermediate range expansion compared with the ataxic phenotype [3–6, 10, 11, 23, 26, 28, 29, 34–36]. In the PD phenotype, expansion numbers were similar, regardless of family history [4, 6, 10, 23, 24, 26–29]. In the MSA phenotype, expansion numbers were both 32 [28, 29]. In addition to the small expansion number of TNR, there is another interesting feature of parkinsonian SCA2: interrupted CAG repeats. Even though some patients with interrupted CAG repeats presented with predominant ataxia [37], all except one case of structurally investigated SCA2-related parkinsonism cases had interruption by CAA, CGG, and CGC [5, 8, 23, 24, 28]. Only one case failed to show interruption, even though that proband had 33 repeats [6]. These interruptions may promote genetic stability and block the formation of higher repetition. Sobczak and Krzyzosiak proposed a hairpin structure for the CAG repeats [38], and they suggested that pure CAG expansion forms a single hairpin arrangement, and interrupted alleles assemble shorter branched hairpin structures, which can affect mRNA transcription or translation. This may explain the low penetrance in SCA2 related parkinsonian cases and why sporadic cases are common.

### 2.4. Pathology

In ataxic SCA2, widespread degeneration with neuronal loss and atrophy of the brain and spinal cord was reported including the brainstem, cerebellum, frontal area, motor cerebello-thalamo-cortical loop, and somatosensory system from Clarke's column to the ventral posterior lateral and ventral posterior medial nuclei of the thalamus [39, 40]. Only two studies have been published on the pathology of parkinsonism with SCA2 [15, 41]. In one report [15], macroscopically, the brainstem, cerebellum, frontal convexity, and spinal cord were atrophic, and the axial sections showed more prominent atrophy at the cerebral peduncle and pontine base. Severe depigmentation was observed in the substantia nigra but not in the locus coeruleus. The other case [41] revealed severe atrophy of the pons, medulla oblongata, and substantia nigra, resembling MSA-cerebellar type. Microscopically, both cases presented widespread antiexpanded polyglutamine antibodies in the neurons including the pontine nucleus, cerebellum, the inferior olivary nucleus, substantia nigra, and frontal cortex. Interestingly enough, there was coexistent Lewy body pathology in the substantia nigra, locus ceruleus, and dorsal motor nuclei of the vagus in both cases [15, 41]. In addition, there were Lewy bodies and neuritis in the sympathetic nerve in the myocardium of one case [41] and in the basal nucleus of the Meynert, hypothalamus, and amygdala in another case [15].

## 3. SCA17

### 3.1. Epidemiology

SCA17 was initially reported by a Japanese group [42] in four Japanese pedigrees with a combination of dementia, ataxia, hyperreflexia, parkinsonism, and other involuntary movements such as dystonia and chorea. Epilepsy was also observed. Abnormal CAG expansion in the TATA-binding protein (TBP) gene with 47 to 55 repeats was found in these families, whereas the normal repeat number ranged from 29 to 42. A case of a 49-year-old man with progressive ataxia, autonomic dysfunction, parkinsonism, supranuclear palsy, and cognitive impairment was reported by a Taiwanese group in 2007 [20]. This case was not a pure parkinsonian phenotype, but it was particularly significant because an 18F-6-fluorodopa PET study showed a marked decrease of fluorodopa uptake in the bilateral putaminal regions and left caudate nucleus [20].

Wu et al. analyzed 334 patients (39 patients with autosomal dominant cerebellar ataxia, 31 patients with sporadic ataxia, and 264 patients with PD); and one patient with dopamine-responsive PD was discovered with a SCA17 expansion with a repeat number of 46 (0.4%) [19]. SCA17 was extensively studied by our group. In a large Korean sporadic parkinsonian population of 1155 patients (931 with PD and 224 with MSA), 0.9% (eight patients with PD and two patients with MSA) were found with SCA17 [21]. In the familial form of parkinsonism, over 7% (two patients out of 27) of the patients showed positive results [21]. Another Korean cohort of sporadic parkinsonism patients (386 with PD and 138 with MSA) had similar results: 0.78% with PD and 2.89% with MSA-P [29].

However, a Singapore cohort failed to discover SCA17-related parkinsonism [26] in 46 familial PD patients and 45 sporadic PD patients. There were no SCA17-related parkinsonian phenotypes in western cohorts neither in the familial nor in the sporadic cases [23, 43].

### 3.2. Clinical Features

Previous reports have presented the heterogeneous clinical features of SCA17 which included cerebellar ataxia with dementia, epilepsy, psychosis, and abnormal movement disorders including chorea, dystonia, and parkinsonism [20, 42, 44]. SCA17 related parkinsonism dominant type revealed similar features with PD. The onset age of PD-mimicking type was from 44 to 75 [19, 21, 29] which is not different from that of PD patients. The PD phenotype is levodopa-responsive and can show motor fluctuation and dyskinesia. We experienced a case with a good levodopa-responsive PD patient with severe motor fluctuation and peak dose dyskinesia who underwent bilateral subthalamic nucleus (STN) Deep Brain Stimulation (DBS) surgery. After DBS, his motor fluctuation and dyskinesia disappeared. Two years later, postural instability developed and mild cerebellar atrophy on the brain MRI was observed [21].

The onset age of MSA-mimicking type was from 54 to 74, and all these MSA patients had the MSA-P phenotype with no levodopa response. Two of them showed no ataxia [21] whereas the other four developed mild ataxia with follow-up [29]. One out of six patients showed putaminal atrophy and two patients showed cerebellar atrophy on the brain MRI [21, 29].

Combinations of other neurological problems with parkinsonism have also been reported. Chorea is a common feature of SCA17, and Huntington's disease-like phenotype has been seen in some of the literature at 0.4 to 0.8% [45–47]. Recently, one study reported reduced dopamine D2 receptor levels in the putamen and caudate of symptomatic SCA17 patients, and many presymptomatic SCA17 patients had already shown reduced D2 levels [22]. Moreover, the D2 levels in the putamen correlated with motor disability level, as assessed by the Unified Parkinson Disease Rating Scale (UPDRS) III.

### 3.3. Genetic Characterization

SCA17 has a vague boundary for the expansion number for the pathologic range. Previous studies suggested the repeat number 43 as a cut-off value [42, 48]. Kim et al. [21] showed the possibility that an expansion as low as 42 repeats could constitute a risk factor or a susceptibility gene for parkinsonism by showing decreased striatal DAT binding in the normal control with 42 repeats. Ataxia patients with only 41 repeats of the TBP gene have also been reported [49–51]. However, there were normal controls with more than 43 repeats. It is still unclear whether 41 repeats could be a risk factor for neurological problems or just an incidental finding. There may exist a modifier that expresses a borderline repeat expansion. Additionally, many patients with SCA17 in structurally investigated studies had CAA interruptions [19, 21, 52, 53], which have been shown in SCA2 related parkinsonism, especially in all the patients with the parkinsonian phenotype [19, 21].

### 3.4. Pathology

Pathologic studies are limited for SCA17. In ataxic SCA17 cases, there was marked atrophy of the cerebellum with the loss of Purkinje cells and mild atrophy in the basal ganglia and cortex [44]. In one patient, substantia nigra atrophy was also observed. Microscopically, intranuclear neuronal inclusion bodies with anti-TBP and 1C2 were widely distributed [44]. Other pathology reports also found similar results including pseudohypertrophic degeneration of the inferior olive, marked neuronal loss, and gliosis in the caudate nucleus and substantia nigra and in the medial thalamic nuclei in 16 affected ataxic patients [54, 55].

However, the pathology for parkinsonian SCA17 has not been studied, and further study is needed in the future.

## 4. SCA3

### 4.1. Epidemiology

SCA3 is the most common SCA worldwide with geographic differences [56] and has been regarded as one of the genetic causes of familial parkinsonism, especially in African ethnicities [57, 58]. A study that described the ethnic differences in the expression between Africans and Caucasians concluded that SCA3 expansion should be considered in the differential diagnosis of all African cases of parkinsonism [58]. There were some familial parkinsonism cases [14, 57] and case series [2, 59] on parkinsonism in SCA3, but only a few cohort studies with large populations [7, 13, 23, 26, 60, 61] were done with only two positive result studies [14, 27]. In a Brazilian population, 7.4% of familial parkinsonism with combined ataxic patients gave a positive result [14]. Wang et al. reported 3% of familial PD and 0.8% of sporadic PD in a mainland Chinese population without ataxic symptoms [27]. A group of 524 Korean patients with parkinsonism (386 with PD and 138 with MSA) was examined for SCA3, but none were found to have SCA3 [29].

### 4.2. Clinical Features

The clinical phenotypes of SCA3 were classically classified into three categories: type 1 with early onset pyramidal and extrapyramidal signs, type 2 with cerebellar and pyramidal symptoms, and type 3 with cerebellar involvement and anterior horn cell degeneration [62]. Parkinsonism with a combination of other neurological symptoms was regarded as the fourth type of SCA3 [2]. A PD-resembling phenotype has been reported in an African-American family with autosomal dominant parkinsonism due to a SCA3 mutation [57]. The CAG repeats of the four patients in the family were 73, 67, 68, and 75. Although they showed cardinal parkinsonian symptoms and levodopa responsiveness, three of them had saccade slowing and one of them had combined peripheral neuropathy. None of them showed ataxia. The PD phenotype in SCA3 with 66 repeats was indistinguishable from PD, including levodopa response and typically expected motor complications in its advanced stages [63].

### 4.3. Genetic Characterization

SCA3 is caused by a CAG expansion in the ATXN3 gene for the protein ataxin-3 [64] with a pathologic expansion number from 52 to 86 [65]. A normal allele has fewer than 44 CAG repeats [66]. Several studies on the genotype and phenotype correlation of SCA3 have been done, but there are no findings on parkinsonism in SCA3. Only the length of the expanded CAG and age at onset showed a strong inverse relationship to each other [62, 67].

### 4.4. Pathology

Many pathology reports have been published about ataxic patients with SCA3 mutations. Macroscopic brain examinations showed the pallor of the substantia nigra as well as the degeneration of the cerebellum and brainstem [68]. Neuronal loss was observed in the cerebral cortex, basal ganglia, thalamus, midbrain, pons, medulla oblongata, cerebellum, and even spinal cord [55, 69, 70]. Chen et al. [71] reported that the degeneration of the subthalamopallidal system was the main neuropathologic features of SCA3. The MSA-C phenotype, which was confirmed by numerous alpha-synuclein-containing glial cytoplasmic inclusions in autopsies, was also reported with 72 repeats for the SCA3 mutation [72]. No autopsy reports for SCA3 patients with the PD phenotype have been reported until now.

## 5. SCA6

SCA6 is from a CAG expansion in the CACNA1A gene [16] generally manifests in the form of pure ataxia [1]. However, there have been some cases with mixed manifestations in SCA6. Kohira et al. presented a case of parkinsonism with ataxia that featured a slow, symmetrical progression and a lack of response to levodopa [73]. Autonomic dysfunction is sometimes observed in SCA6. Lee et al. reported two cases of parkinsonism with urinary incontinence in non-juvenile-onset parkinsonism with the SCA6 mutation, which were misdiagnosed as MSA [74]. Korean data showed that the striatal DAT density is variably reduced in SCA6 [18]. Yun et al. reported on a patient with young onset parkinsonism without cerebellar dysfunction who showed no improvement with levodopa at 800 mg/day [75]. His expansion number for SCA6 was increased to 20 (less than 16 in a normal population) [76]. Gastric cancer was found during the follow-up, and immunohistochemistry in the resection margin from his stomach showed no alpha-synuclein-positive inclusions.

## 6. SCA8

The SCA8 mutation involves two overlapping genes ATXN8OS and ATXN8 [67] with normal alleles of 15 to 50 CTG repeats; it is mainly characterized by cerebellar involvement with hyperactive tendon reflexes [77]. Wu et al. detected an abnormal expansion of SCA8 in four patients with typical PD (1.5%) from among 264 patients with PD [19]. The range of the SCA8 repeat size was analyzed in a Taiwanese PD cohort, and large SCA8 alleles (66–120 repeats) and a novel ATXN8 −62 G/A promoter SNP were found [78]. The same group also performed a structural analysis in a cohort of 569 PD cases and 547 ethnically matched controls, and they found that individuals carrying the AA genotype exhibited a decreased risk of developing PD than those with the GG + GA phenotypes [71]. A Japanese group analyzed the SCA8 CTA/CTG repeat for 2806 people including 448 PD patients, and 0.4% had expanded alleles (85–399) while there were no individuals with expansion among the 654 normal controls [79]. A patient with levodopa-responsive parkinsonism with additional movement disorders such as a dystonic gait and an unusual oscillatory movement of the trunk was reported as having a mutation in SCA8 in Korea [80].

## 7. Conclusion

SCA can present as parkinsonism, especially in SCA2, SCA3, and SCA17. SCA3 is more common in western populations, and SCA2 and SCA17 are more prevalent in Asian populations. SCA6 and SCA8 may also present parkinsonism in some cases. The important thing is that SCA2 and SCA17 may very closely mimic PD and be a not uncommon genetic cause of parkinsonism in Asian regions even in sporadic cases. Thus, the screening of SCA2, SCA3, and SCA17 may be required in PD patients. Small expansion with interruption could explain the parkinsonian phenotype and sporadic cases with low penetrance. A direct interaction between alpha-synuclein accumulation and a shorter expansion of CAG repeats are under investigation. The association between amyotrophic lateral sclerosis and ATXN2 has been known as an increased risk [81–83], and the coexistence of SCA2 and ALS was also reported [84]. This implies that SCA can involve not only the cerebellar system but also other nervous systems and cause diverse neurodegenerative diseases.

In conclusion, even when a patient shows parkinsonism alone, we need to consider that SCA could be the differential diagnosis. There is a need for careful pathological examination to explain why SCA can present as parkinsonism. Furthermore, why there are geographical or ethnic differences in SCA related parkinsonism which needs to be investigated.

## Figures and Tables

**Table 1:** Literature review: clinical and genetic characteristics of SCA2, SCA3, and SCA17 with parkinsonism.

Study	Country	Parkinsonism type	Prevalence, %	Number of study population	Number of affected patients	Onset age, y	Expansion number of patients	Levodopa response	Cerebellar signs	Noticeable clinical features	Long term follow-up complications
SCA2											
Shan et al. (2001) [4]	Taiwan	Familial	8.69	23 patients in 19 families	2	50/50	36/37	+	−	Both tremor dominant with slow saccade and CIT-PET (+), nystagmus (+) in 1 patient	
Kock et al. (2002) [85]	Mixed	Familial DRP	0	64 young onsets, 32 late onsets (age of onset >50)	0						
Payami et al. (2003) [6]	USA	Familial DRP	1.5	136	2	36 (Caucasian)/60 (Hispanic)	33/35	+	−	Evidences of peripheral neuropathy (+) in 1 patient	(1) D+, MF+
Svetel et al. (2003) [86]	Serbia	Familial YO-DRP	0	40	0						
Lu et al. (2004) [10]	Taiwan	Familial	5.38	130 patients in 41 families	7	45.8 ± 13.9	35–38	+	+^a^	1 patient with mild slow saccades and 1 patient with combined mild dystonia	D+^c^
Simon-Sanchez et al. (2005) [13]	USA	Familial	0.88	114	1	55 (Caucasian)	37	+			
Lim et al. (2006) [26]	Singapore	Familial IPD	0	46	0						
Charles et al. (2007) [23]	France	Familial ADP	2	178 patients in 164 families	3	29–64 (50.1 ± 13.2)^d^	37–39^d^	+	−		D 22%, MF 14%^d^
Lin et al. (2007) [60]	Taiwan	Familial	0	13	0						
Modoni et al. (2007) [24]	Italy	Familial	2.5	79	1	48	38	+	−	Tremor dominant and CIT-PET (+)	D−, MF−
Wang et al. (2009) [27]	China	Familial PD	1.5	66	2	42/35	36/36	+	−	CIT-PET (+)	

Kock et al. (2002) [85]	Mixed	Sporadic DRP	0	174	0						
Svetel et al. (2003) [86]	Serbia	Sporadic YO-DRP	0	45	0						
Shan et al. (2004) [11]	Taiwan	Sporadic PD and atypical parkinsonism	0.4	242	1 in PD group and 0 in atypical group	56	37	+	−	CIT-PET (+)	D+, MF+
Lim et al. (2006) [26]	Singapore	Sporadic YO-IPD	2.2	45	1	50	36	+	−	Postural instability	
Tan et al. (2007) [61]	Singapore	Sporadic atypical parkinsonism patients with poor levodopa response	0	100	0						
Lin et al. (2007) [60]	Taiwan	Sporadic	0	60	0						
Modoni et al. (2007) [24]	Italy	Sporadic	0	145	0						
Kim et al. (2007) [28]	Korea	Sporadic PD	0.43	468	2	70/55	35/34	+	−	Saccadic movement dysfunction, hyporeflexia in 1 patient	
Kim et al. (2007) [28]	Korea	Sporadic MSA-P	0.74	135	1	59	32	Minimal	+	MSA-P type MRI: cerebellar atrophy	
Wang et al. (2009) [27]	China	Sporadic	0.5	386	2	29/37	37/36	+	−		
Yun et al. (2011) [29]	Korea	Sporadic PD	0	386	0						
Yun et al. (2011) [29]	Korea	Sporadic MSA	0.72	138	1	55	32	−	+	MSA-P type CIT-PET (+) MRI: cerebellar atrophy	

SCA3											
Svetel et al. (2003) [86]	Serbia	Familial YO-DRP	0	40	0						
Simon-Sanchez et al. (2005) [13]	USA	Familial	0	114	0						
Lim et al. (2006) [26]	Singapore	Familial PD	0	46	0						
Lin et al. (2007) [60]	Taiwan	Familial	0	13	0						
Charles et al. (2007) [23]	France	Familial ADP	0	178 patients in 164 families	0						
Wang et al. (2009) [27]	China	Familial	3	66	4	46/25/40/39	65/69/73/67	+	+^b^	CIT-PET (+)	
Svetel et al. (2003) [86]	Serbia	Sporadic YO-DRP	0	45	0						
Lim et al. (2006) [26]	Singapore	Sporadic YO-IPD	0	45	0						
Lin et al. (2007) [60]	Taiwan	Sporadic	0	60	0						
Tan et al. (2007) [61]	Singapore	Sporadic atypical parkinsonism patients with poor levodopa response	0	100	0						
Wang et al. (2009) [27]	China	Sporadic	0.8	386	3	67/36/38	58/64/67	+	−		
Yun et al. (2011) [29]	Korea	Sporadic PD	0	386	0						
Yun et al. (2011) [29]	Korea	Sporadic MSA	0	138	0						

SCA17											
Hernandez et al. (2003) [43]	USA	Familial	0	51	0						
Lim et al. (2006) [26]	Singapore	Familial PD	0	46	0						
Charles et al. (2007) [23]	France	Familial ADP	0	178 patients in 164 families	0						
Kim et al. (2009) [21]	Korea	Familial PD	7.41	27	2	44/50	43/45	+	−		D+, MF+
Hernandez et al. (2003) [43]	USA	Familial	0	59	0						

Wu et al. (2004) [19]	Taiwan	Sporadic PD	0.38	264	1	75	46	+	−		
Lim et al. (2006) [26]	Singapore	Sporadic YO-IPD	0	45	0						
Kim et al. (2009) [21]	Korea	Sporadic PD	0.66	904	6	Mean 53.75 (44–61)	43/44	+	−		
Kim et al. (2009) [21]	Korea	Sporadic MSA	0.89	223	2	61/54	29–46	−	+		
Yun et al. (2011) [29]	Korea	Sporadic PD	0.78	386	3	47/66/62	45/44/43	+			
Yun et al. (2011) [29]	Korea	Sporadic MSA	2.89	138	4	54/59/74/62	46/44/43/43	Partial	+	MSA-P type	

^a^Mild dysarthria, ataxic gait, and postural instability in the late stage.

^b^Positive in 2 patients.

^c^Positive in 1 patient.

^d^Prevalence in total 9 including affected family members.

DRP: dopa-responsive parkinsonism, YO: young onset, IPD: idiopathic Parkinson disease, ADP: autosomal dominant parkinsonism, MSA-P: parkinsonian variant of multiple system atrophy, D: dyskinesia, and MF: motor fluctuation.

Each patient is separated with slash mark in onset age and expansion numbers.
